# The Use of Curcumin as a Complementary Therapy in Ulcerative Colitis: A Systematic Review of Randomized Controlled Clinical Trials

**DOI:** 10.3390/nu12082296

**Published:** 2020-07-31

**Authors:** Mariana Roque Coelho, Marcela Diogo Romi, Daniele Masterson Tavares Pereira Ferreira, Cyrla Zaltman, Marcia Soares-Mota

**Affiliations:** 1 Institute of Nutrition Josué de Castro, Federal University of Rio de Janeiro, 21941-971 Rio de Janeiro, Brazil; mariana_coelho_7@hotmail.com (M.R.C.); cela_romi@hotmail.com (M.D.R.); 2 Library of Health Sciences Center, Federal University of Rio de Janeiro, 21941-971 Rio de Janeiro, Brazil; danimasterson@yahoo.com.br; 3 Division of Gastroenterology, Department of Internal Medicine, Federal University of Rio de Janeiro, 21941-913 Rio de Janeiro, Brazil; c.zaltman@gmail.com

**Keywords:** inflammatory bowel disease, proctocolitis, turmeric, curcumin, complementary therapies, phytotherapy

## Abstract

The objective of this study was to systematically review the literature to verify the efficacy and safety of curcumin as a complementary therapy for the maintenance or induction of remission in patients with inflammatory bowel disease (IBD). A comprehensive search was conducted by two independent authors in MEDLINE (PubMed), Scopus, Web of Science, the Cochrane Library, Lilacs, Food Science and Technology Abstracts, and ScienceDirect. The search terms “curcumin”, “curcuma”, “inflammatory bowel disease”, “proctocolitis”, “crohn disease”, and “inflammation” were combined to create search protocols. This study considered randomized controlled trials (RCTs) published in any language before March 2020 that evaluated the effects of curcumin on inflammatory activity and the maintenance or remission of IBD patients. After duplicates were removed, 989 trials were identified, but only 11 met the eligibility criteria. Five of these were considered to be biased and were excluded. Therefore, six trials were considered in this review. All the studies included in the systematic review were placebo-controlled RCTs conducted on individuals with ulcerative colitis (UC). All the RCTs reported that curcumin was well tolerated and was not associated with any serious side effects. Studies show that curcumin may be a safe, effective therapy for maintaining remission in UC when administered with standard treatments. However, the same cannot be stated for Crohn’s disease due to the lack of low bias risk studies. Further studies with larger sample sizes are needed before curcumin can be recommended as a complementary therapy for UC.

## 1. Introduction

Inflammatory bowel disease (IBD) is a chronic condition that affects the relapsing gastrointestinal tract, with periods of exacerbation and remission [1,2]. Its main forms of presentation are ulcerative colitis (UC) and Crohn’s disease (CD). Its etiopathogenesis is believed to be due to a loss of tolerance to the intestinal microbiota associated with marked immune responses and environmental factors in genetically susceptible individuals [3].

The conventional approach to IBD aims to induce and maintain clinical remission free of corticosteroids, thus minimizing the impact on quality of life [4]. Currently, corticosteroids, sulfasalazine, mesalamine (5-ASA), and immunomodulators are treatment options for patients with IBD. However, it is worth mentioning that conventional treatments cause numerous side effects due to a marked immune response suppression, which negatively impacts the quality of life of these individuals [5,6]. Studies indicate that a substantial proportion of patients do not fully respond to the conventional treatments for IBD, or that its efficacy wanes over time [7]. Corticosteroid resistance/refractoriness rates range from 8.9% to 25% in individuals with IBD [8,9,10,11].

Identifying safe and effective therapeutic agents for complementary therapies remains an unmet need for these patients. *Curcuma longa* is a plant from the Zingiberaceae family that is native to India and Southeast Asia and is well known in Asian cultures. Known commonly as turmeric, it has long been used in Ayurvedic medicine to treat inflammatory diseases. It has attracted the attention of researchers because of its compounds, called curcuminoid pigments, which are polyphenols with important medicinal properties [12,13].

Curcumin is the main pharmacologically active curcuminoid pigment in turmeric. It acts by modulating various cell-signaling pathways, producing anti-inflammatory, anti-tumor, anti-oxidant, and immunomodulatory effects [14]. The main components of commercial turmeric are curcumin I (77%), curcumin II (~17%), and curcumin III (~3%), with only 2–5% of the powdered seasoning consisting of curcumin [15]. The mechanism of its anti-inflammatory action deemed to be the most relevant is the inhibition of NF-κB, by blocking IκB kinase, which prevents cytokine-mediated phosphorylation and the degradation of IκB, an NF-κB inhibitor, thereby inhibiting the expression of pro-inflammatory cytokines (IL-1, IL-6, and TNF) [16].

Curcumin also acts by inhibiting the activity of pro-inflammatory proteins such as activated protein-1, peroxisome proliferator-activated receptor gamma, signal translators, and transcription activators, as well as the expression of b-catenin, cyclooxygenase 2, 5-lipoxygenase, and inducible nitric oxide synthase isoform, which play a key role in inflammation [17]. In addition, it acts by blocking the binding between TNF-α and its receptor, preventing the perpetuation of inflammation caused by this cytokine [18].

This systematic review aims to analyze the studies published so far, to review the positive or negative effects of the use of curcumin, and to determine whether it is safe and effective as a complementary therapy in the management of IBD, offering fewer side effects than conventional therapies. 

## 2. Methods

### 2.1. Protocol and Registration

To conduct the study, we used the PRISMA checklist, composed of 27 items [19]. The study protocol was registered in the PROSPERO database under the registration number CRD42019104827.

### 2.2. Information Sources and Search Strategies

A literature review was performed by two independent authors (M.R.C. and M.D.R.) on the following databases: MEDLINE (PubMed), Scopus, Web of Science, Cochrane Library, Lilacs, Food Science and Technology Abstracts, and Science Direct. Studies published before March 2020 were included. All the databases were monitored periodically until the study’s completion. Divergences between the researchers retrieving the data were resolved by consensus.

The controlled vocabulary and keywords used in the search strategy were defined based on the PICOS questions [20]:

1. Population: individuals with IBD (UC or CD) of either sex and from any age group;
2. Intervention: curcumin supplementation in the form of spice, capsule, or enema;
3. Comparison: placebo or conventional drug therapy;
4. Outcomes: disease activity, clinical, or endoscopic inflammatory activity;
5. Study design: randomized clinical trials (RCTs).

The search strategy was designed following the guidance of an expert librarian (D.M) and according to the specificity of each database, whenever possible, using the controlled vocabulary of the subject descriptors (Mesh/Medline and DeCs/VHL). The following subject headings and free-text terms were used in the search: “curcumin”, “curcuma”, “inflammatory bowel disease”, “proctocolitis”, “crohn’s disease”, and “inflammation” (Table 1).

### 2.3. Eligibility Criteria

The criteria for the inclusion of the RCTs in this study were that they used curcumin for the maintenance or remission of IBD in patients of both sexes and of any age who were in remission or who had mild or moderate activity at the time of recruitment, and that they evaluated the effects of curcumin on the inflammatory activity. Studies published in any language were accepted, and no minimum follow-up period was established. Review articles, animal studies, editorial letters, in-vitro studies, observational, and descriptive studies, such as case reports and case series, were excluded. In addition, studies that did not describe the curcumin dose or did not meet the minimum bias risk assessment score were also excluded.

### 2.4. Study Selection and Data Collection Process

Initially, the articles were selected by title and abstract. Articles that appeared in more than one database were considered only once, using the EndNote bibliography management software to exclude duplicate articles. Full articles were read when there was not enough information in the title and abstract to make a clear decision about whether to include or exclude the study.

### 2.5. Risk of Bias Assessment

Two independent reviewers performed the quality assessment of the trials using the Cochrane Collaboration tool for assessing the risk of bias in RCTs [21]. There were seven assessment criteria: random sequence generation; allocation concealment; blinding of participants and personnel; blinding of the outcome assessors; incomplete outcome data; selective outcome reporting, and other possible sources of bias. The potential risk of bias for each criterion was rated at low, uncertain, or high, as described in the *Cochrane Handbook for Systematic Reviews of Interventions*, version 5.1.0 (http://handbook.cochrane.org). Studies with a high risk of bias in three or more items were excluded from the systematic review.

The Oxford quality scoring system, the Jadad scale [22], was also used to assess the study quality. This scale provides a score for each individual study ranging from 0 to 5 points, with 5 being the highest quality score. Studies were given one point if they were described as randomized, one if they were described as double-blind, and one if a description of the withdrawals and dropouts from the study was provided. An additional point was awarded if the randomization method was described and considered appropriate, and another point if the blinding method was described and also considered appropriate. If any of the randomization or blinding methods were considered inappropriate, a point was deducted from the sum for each item.

Each criterion was judged by one of three answers: “yes” to indicate a low risk of bias, “no” to indicate a high risk of bias, and “not described” to indicate a lack of information or uncertainty about potential bias. Studies with scores < 3 were considered to have a high risk of bias and were excluded from the study, while scores ≥ 3 indicated studies with a low risk of bias, which were retained in the analysis [22].

## 3. Results

### 3.1. Search Results

**Figure 1:**
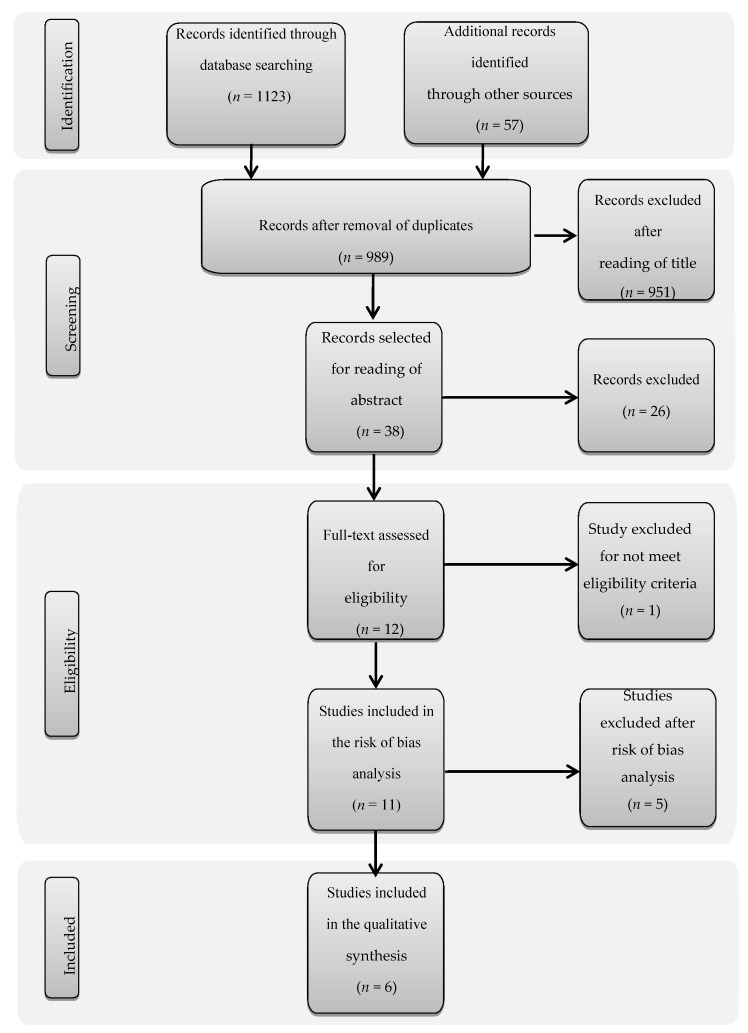
Flowchart of articles selected for the systematic review.

After database screening and duplicate removal, 989 studies were identified (Figure 1). The title analysis resulted in the exclusion of 951 of these, while a further 26 were excluded after reading the abstracts because they failed to meet the eligibility criteria. The twelve remaining articles were read in full and only one of these was ruled out as it was a case report. Eleven studies were included in the bias risk analysis, after which only six remained in this systematic review.

### 3.2. Assessment of the Risk of Bias and Excluded RCTs

After assessing the risk of bias, two studies [23,24] were excluded because they presented three items with a high risk of bias, as recommended in the Cochrane Handbook [21], and did not reach the minimum score necessary on the Jadad scale [22]. The three other studies [25,26,27], excluded at this point, also failed to reach the minimum score on the Jadad scale [22] and had four or more items classified as uncertain according to the Cochrane Handbook [21] because they did not have enough information for the analysis, which put them at a high risk of bias.

**Figure 2:**
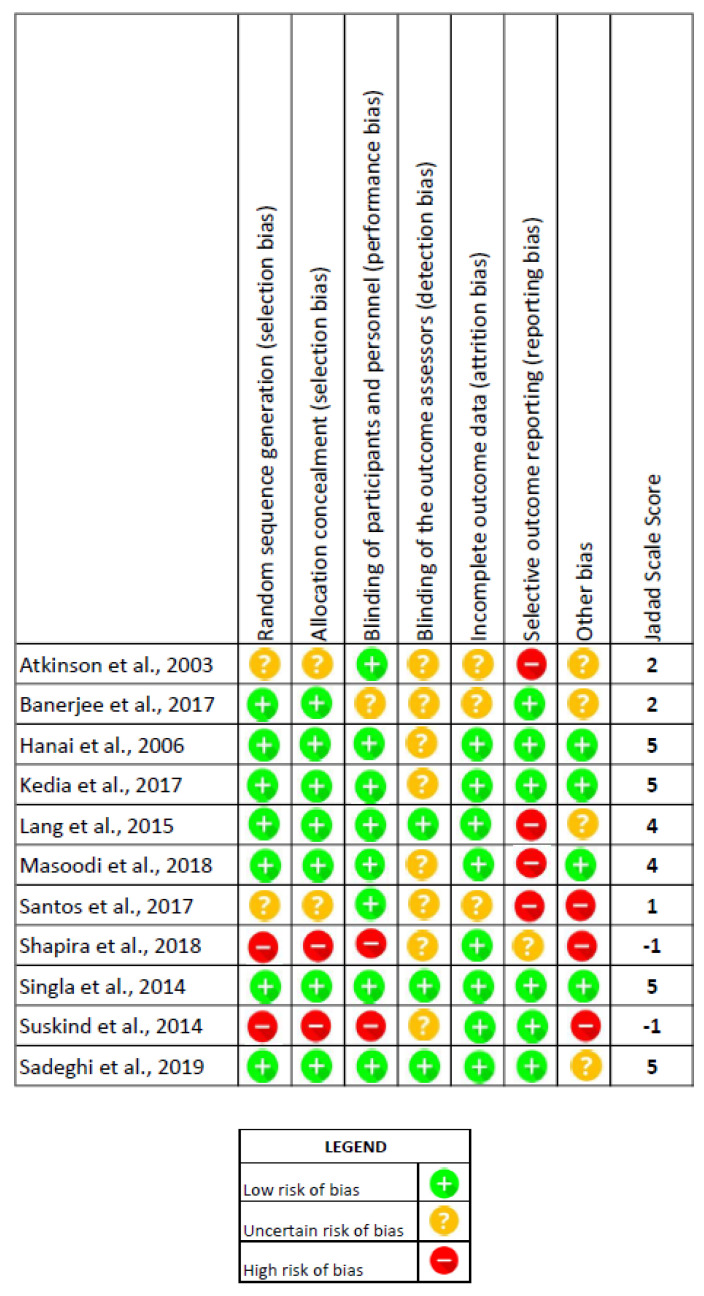
Bias risk assessment according to the Cochrane Handbook tool and the Oxford Quality Scoring System, the Jadad Scale.

The assessment of the risk of bias in the selected studies is presented in Figure 2. Most of the articles clearly described the randomization method used [26,28,29,30,31,32,33], as well as the blinding method [26,27,28,29,30,31,32,33]. Some studies [25,26,27] reported incomplete outcome data and others [27,29,32,33] presented the outcomes selectively, which indicates a description bias. With regard to the allocation concealment, two studies [25,27] did not describe this and two others [23,24] stated that there was none.

All the studies included in the systematic review presented the main elements recognized to minimize the risk of bias, according to the Cochrane Handbook [21]: randomization, blinding, and reporting of dropouts/withdrawals.

### 3.3. Characteristics of Selected Articles

All six of the studies included in the systematic review were placebo-controlled RCTs performed on individuals with UC and conducted between 2006 and 2019. No studies involving CD met all the eligibility criteria. Table 2 details the characteristics and results of each study. They were all conducted in outpatient settings, including a total of 372 subjects, ranging in age from 23 to 61 years. There were no significant differences regarding the number of male and female patients who participated in these trials.

The studies compared the use of curcumin as a complementary therapy given in combination with mesalamine (5-ASA), a conventional drug regularly prescribed for patients with UC, with placebos also in conjunction with 5-ASA. The oral capsule curcumin dosage ranged from 450 mg to 3 g/day. One study reported using NCB-02, a standardized extract with 72% curcumin, 18.08% demethoxicurcumin, and 9.42% bisdemetoxicurcumin as an enema at a dosage of 140 mg/day [31]. One study used capsules of a nanomicellar curcumin formulation (SinaCurcumin^®^) at doses of 240 mg/day [33]. The duration of the interventions ranged from 4 weeks to 12 months.

Different methods were used to evaluate the clinical activity of the disease, but in general the parameters evaluated by the different scores included: number of bowel movements, fecal urgency, bloody stools, self-reported general well-being, abdominal pain, and extra-intestinal manifestations. The endoscopic scores used evaluated the following parameters: vascular pattern, presence of erythema, friability of the mucosa, erosions, spontaneous bleeding and presence of ulcerations.

### 3.4. Outcomes after Intervention

In most of the studies, positive outcomes were reported after the interventions. Hanai et al. (2006) reported a lower number of relapses in the intervention group than in the control group, while other studies showed a higher proportion of remission in the intervention group [29,30]. Significant clinical responses measured through the disease activity indices and an improved endoscopic activity were also reported in four of the five studies, except for the study conducted by Kedia et al. [32], which showed no significant difference between the clinical and endoscopic remission rates of the intervention and placebo groups.

In Masoodi’s study [31], in addition to a significant reduction in the simple clinical colitis activity index (SCCAI), a higher proportion of the intervention group patients reported improved general well-being and decreased fecal urgency than did the patients from the placebo group after four weeks. Curcumin was well tolerated in all the RCTs and was not associated with any serious side effects. However, Hanai et al. [28] did report some mild adverse events (AE), such as: feeling of abdominal distension, nausea, and a transient increase in the number of bowel movements.

The only one to use a quality of life questionnaire [33] showed a significant increase in the mean score of IBDQ-9 in the curcumin group compared to the placebo group. In the same study, curcumin supplementation induced a significant reduction in the high-sensitivity C-reactive protein (hs-CRP) concentrations after eight weeks vs. no significant reduction seen in the placebo group.

## 4. Discussion

This systematic review included six RCT studies [28,29,30,31,32,33] that compared the use of curcumin as a complementary therapy given in combination with 5-ASA in patients with UC. In four of the five studies included, there was a significant improvement in the clinical response with curcumin, allied with no serious AEs. Five studies [28,29,30,31,33] reported that curcumin was able to reduce the symptoms of the disease, achieve clinical remission, and/or prevent relapse when used as a complementary therapy to mesalamine.

The study by Kedia et al. [32], using lower doses of oral capsule curcumin than other studies [28,30,33], which used 2000 mg/day, 3000 mg/day and 1500 mg/day, respectively, was the only study in which no significant difference was found between clinical remission and the endoscopic remission rates in the intervention and placebo group (UCDAI). It is understood that very low doses of curcumin may not achieve the desired effect unless administered locally in the form of an enema, as in the study by Singla et al. [29], or in more bioavailable nanoformulations such as SinaCurcumin^®^, used in the study by Masoodi et al. [31].

The curcuminoid gelatin capsule is dissolved in the acidic environment of the stomach and the nanomicelles are released, which are stable for up to 6 h and are absorbed into the intestine [34]. Importantly, curcumin absorption may be lower when it is administered orally than when it is administered in an enema when it comes to IBD, due to the direct delivery to the site-of-action. Therefore, RCTs should also explore the enema administration of curcumin in order to define the most effective route of administration, since only one RCT [29] has evaluated this route so far.

Most of the studies did not describe the purity of the curcumin used. Singla et al. [29] only described the percentage of curcumin in the extract used, while Lang et al. [30] reported using Cur-Cure^®^ (a preparation containing 95% pure curcumin); the other four studies did not provide any such information. Any comparison of this study with others that do not describe the composition of the capsule is hampered by the fact that it is not known exactly what the intervention groups in the other studies were given. An important caveat is that capsule curcumin found on pharmaceutical and nutritional store shelves often contains numerous chemical additives, as does the turmeric powder sold commercially as a spice.

Only Hanai et al. reported some mild AEs, such as abdominal distension, nausea and an increased number of bowel movements in a Japanese population. These non-specific symptoms may be related to factors not controlled by the researchers, such as dietary factors (lactose, fodmaps, gluten) and the presence of associated functional diseases, such as IBS (irritable bowel syndrome). Therefore, it cannot be confirmed that these symptoms experienced by only 7 of 89 patients are related to curcumin use.

The Food and Drug Administration states that curcumin is “generally recognized as safe” and has no known toxic effects. According to the Food and Agriculture Organization of the United NationsFAO/WHO Joint Food Additives Expert Committee and the European Food Safety Authority, the acceptable daily intake of curcumin is 0–3 mg/kg/day [35,36]. Lao et al. [37] administered 500–12,000 mg of curcumin (95% standardized extract of curcumin) in healthy subjects to examine its maximum tolerance and safety dose, and found that an ingestion of up to 12 g/day of curcumin brought about no ill effects. Based on this recommendation, a healthy 70 kg individual could consume 4–10 g turmeric powder, or two tablespoons, per day, which is well above the usual consumption in western countries.

The bioavailability of orally ingested unformulated curcumin is low. High doses of curcumin (2–4 g) are usually required to improve bioavailability due to its hydrophobic nature, but recently, in addition to nanoformulations, studies have been conducted into a self-micro emulsifying drug delivery system (SMEDDS) for curcumin: hydrophilic drug droplets that can diffuse easily into the bloodstream, resulting in higher intraintestinal concentrations than when conventional curcumin is used [26]. Although SMEDDS has been used successfully in a study, the current evidence on its usefulness is still scant. In a single-blind crossover study on healthy adults, the bioavailability of the curcumin micellar formulation was found to be 185 times higher than that of the same dose of unformulated curcumin [38]. Despite promising results concerning curcumin micellization, the study was conducted on healthy subjects; there are no studies on individuals with IBD. This nanometer-sized drug delivery system could become an effective strategy for treating IBD [39].

In a recent systematic review, Jamwal [40] compared the pharmacokinetic effect of different curcumin formulations in healthy subjects. The formulations that exhibited a better bioavailability than unformulated curcumin were: NovaSol^®^, CurcuWin^®^, and LongVida^®^, which were found to achieve a 185, 136, and 100 times higher bioavailability, respectively. The studies cited report promising preliminary results from in vitro and in vivo experimental trials by developing specific curcumin delivery systems, protecting against rapid degradation, and targeting the inflamed colon. However, it is not yet known which would be the best option from those among the most bioavailable formulations for treating IBD.

Another strategy for improving bioavailability, the use of piperine (a component of black pepper, *Piper nigrum*, and long pepper, *Piper longum*) as an adjunct to curcumin, has been described for a long time. Shoba et al. [41] demonstrated that 20 mg of piperine administered concomitantly with 2 g of curcumin increases the bioavailability of curcumin 20-fold in humans. One experimental study by Li et al. [42], using CUR-PIP-SMEDDS (an emulsified curcumin and piperine formulation), reported that the administration of this formulation through an enema in rats had an effect similar to 5-ASA in maintaining remission in dextran sulfate sodium DSS-induced colitis. As a substance that inhibits the metabolism of several active compounds, piperine may cause toxicity when associated with some drugs [43]. Moreover, the gastrointestinal transit time has been reported to be significantly reduced by the consumption of 20 mg of piperine by rats [44], which in individuals with IBD may not be beneficial.

Despite the small number of studies, the average intervention time that appears to be sufficient to present the outcomes is four to eight weeks. However, this time may vary according to the pharmaceutical formulation used. Shorter intervention periods seem to be associated with more bioavailable curcumin formulations.

It is noteworthy that phytochemicals are not evenly distributed among plant parts: the curcumin content of mother rhizomes is higher than the curcumin content of primary and secondary rhizomes, both fresh and dried [45]. The seasonality, altitude, temperature, solar incidence, water availability, and nutrient content in the soil may also alter the presence or concentration of phytochemicals [46]. Post-harvest processing for the production of dry powder results in a reduced curcumin content in the mother’s rhizomes and fingers, as it is easily decomposed when exposed to light and is sensitive to high temperatures (>60 °C) [47].

In addition, the pre-harvest management, specifically cultivation practices, also play a significant role in the quantity and quality of turmeric phytochemicals. In the study by Choudhury et al. [47], the application of nitrogen, phosphorus, potassium (NPK)inorganic fertilizers resulted in a 31–43% increase in the curcumin content of the mother rhizome compared to the non-application of NPK fertilizers. Similarly, the application of organic fertilizers (pig and poultry manure) also increased the curcumin content of mother rhizomes by 18–36%. All the factors mentioned may have influenced the curcumin content used in the studies and, consequently, the results obtained.

Efforts are currently being made by different research groups to improve curcumin’s bioavailability, and further efforts will be needed to answer questions related to curcumin therapy in IBD. New well-designed, long-term RCTs with a large enough sample size to demonstrate clinically significant effects and to determine the efficacy of new pharmaceutical formulations are required.

Considering the six trials included, five of them demonstrated good results related to the clinical and/or endoscopic remission/response. The findings suggest that curcumin may be a safe, effective therapy for maintaining or inducing UC remission when administered with standard treatments; the same cannot be said for CD, due to the absence of RCTs with a low risk of bias when investigating patients with this condition. However, based on these findings it is not yet possible to establish the best protocol for the use of curcumin, considering that the determination of the therapeutic dose and the duration of treatment depend on the identification of the ideal pharmaceutical formulation, which has not yet been determined for this population.

We recognize that this study inevitably has some limitations and so its results should be interpreted with caution. Firstly, it was based on only six RCTs, all with a relatively small sample size. Additionally, although the studies included were well designed, randomized, placebo-controlled studies that rated highly on the Jadad scale [22] and in the Cochrane Handbook bias analysis [21], they used different doses and formulations of curcumin and different administration routes. This itself may add bias to the results of our study.

## Figures and Tables

**Table 1:** Electronic Databases and Respective Search Strategies.

**PubMed**	
**#1 (Inflammatory Bowel Disease [Mesh] or Inflammatory Bowel Disease [Tiab] or Crohn Disease [Mesh] or Crohn Disease [Tiab] or Proctocolitis [Mesh] or Proctocolitis [Tiab])**	#2 (Curcuma [Mesh] or Curcuma [Tiab] or Curcumin [Mesh] or Curcumin * [Tiab])
#1 AND #2	
**Scopus**	
#1 (TITLE-ABS-KEY ((“Inflammatory Bowel Disease” or “Crohn Disease” or proctocolitis)))	#2 (TITLE-ABS-KEY ((curcuma or curcumin *)))
#1 AND #2	
**Web of Science**	
#1 (“Inflammatory Bowel Disease” or “Crohn Disease” or Proctocolitis)	#2 (Curcuma or Curcumin *)
#1 AND #2	
**Lilacs**	
#1 tw: (tw: ((mh: “inflamatory bowel diseases” or “doenças inflamatórias intestinais” or mh: “crohn disease” or “doença de crohn” or mh: proctocolitis or “retocolite ulcerativa”)))	#2 (tw: (tw: ((mh: curcumin or curcumina or curcuma))))
#1 AND #2	
**Food Science and Technology Abstracts**	
#1 (“Inflammatory Bowel Disease” or “Crohn Disease” or Proctocolitis)	#2 (Curcuma or Curcumin *)
#1 AND #2	
**ScienceDirect**	
#1 (“Inflammatory Bowel Disease” or “Crohn Disease” or Proctocolitis)	# (curcumin or curcumina or curcuma)
#1 AND #2	
**Cochrane Library**	
#1 MeSH descriptor: [Inflammatory Bowel Diseases] explode all trees	#8 Proctocolitis
#2 “Inflammatory Bowel Disease”	#9 #7 or #8
#3 #1 or #2	#10 #3 or #6 or #9
#4 MeSH descriptor: [Crohn Disease] explode all trees	#11 MeSH descriptor: [Curcuma] explode all trees
#5 “Crohn Disease”	#12 (Curcuma or Curcumin or Curcumin *)
#6 #4 or #5	#13 #11 or #12
#7 MeSH descriptor: [Proctocolitis] explode all trees	#14 #10 and #13

#- represents the combination of searches conducted previously; *- matches one or more occurrences of any character or group of characters, including no character.

**Table 2:** Summary of studies included in the systematic review.

Author, Year and Country	Study Design	Characterization of UC Population	Intervention	Variables of Interest Analyzed	Results
Hanai et al. 2006 [28]	Multicenter, randomized, double-blind, placebo-controlled	In remission	Curcumin (capsule) 2 g + 1.5–3 g 5ASA or 1–3 g sulfasalazine/day (*n* = 45) or placebo + 5ASA/sulfasalazine (*n* = 44)	Clinical Activity Index and Endoscopic Index assessed at baseline and every 2 months up to 12 months	7 participants did not complete the protocol. 2/43 (4.65%) curcumin group and 8/39 (20.51%) placebo group relapsed at 6 months. Curcumin significantly improved Clinical Activity Index and Endoscopic Index. Curcumin was well tolerated and not associated with any SAE.
		*n* = 89 (49♂/30♀)			
Japão		25–61 years	6 months		
Singla et al. 2014 [29]	Pilot study, double-blind, randomized, placebo-controlled	Mild/moderate proctitis and proctosigmoiditis	140 mg NCB-02 (standardized extract curcumin) enema + oral 1.6 g 5ASA/day (n = 28) or placebo enema + oral 1.6 g 5ASA/day (*n* = 22)	UCDAI and endoscopic activity by mucosal appearance score at baseline and after 8 weeks	9 NCB-02 group and 6 placebo group did not complete the protocol. At the end of 8 weeks, Clinical response: 13/14 (92.9%) NCB-02 vs. 50% placebo group. Clinical remission: 71.4% NCB-02 vs. 31.3% placebo group. Improvement of Endoscopic activity: 85.7% NCB-02 vs. 50% placebo group. No SAE.
		*n* = 45 (22♂/23♀)			
India		23–49 years	8 weeks		
Lang et al. 2015 [30]	Multicenter, randomized, double-blind, placebo-controlled	Mild/moderate proctitis/left colitis/pancolitis	95% pure curcumin (capsule)—3 g + 4 g 5ASA/day (*n* = 26) or placebo + 4 g 5ASA/day (*n* = 24)	SCCAI and Mayo endoscopic score assessed at baseline and after 4 weeks	2 participants did not complete the protocol. Clinical remission (SCCAI): 54% curcumin vs. 0% placebo group. Clinical response: 65.3% curcumin vs. 12.5% placebo group. Endoscopic remission (Mayo): 38 curcumin vs. 0% placebo group.
		*n* = 50 (17♂/33♀)			
Israel, Hong Kong and Cyprus.		27–55 years	4 weeks		
Masoodi et al. 2018 [31]	Single-center, Double-blind, randomized, placebo-controlled	Mild/moderate left colitis/pancolitis	Nanomicellar curcumin (capsule) 80 mg 3x/day = 240 mg + 3 g 5ASA/day (*n* = 28) or placebo + 3 g 5ASA/day (*n* = 28)	SCCAI assessed at baseline, and at 2 and 4 weeks	2 participants did not complete the protocol. Significant reduction of fecal urgency: 60% curcumin vs. 28.6% placebo group. Improvement of general well-being: 64% curcumin vs. 39.3% placebo group). Significant reduction in SCCAI score (1.71 points curcumin group vs. 2.68 points placebo group).
		*n* = 56 (28♂/28♀)	4 weeks		
Iran		25–54 years			
Kedia et al. 2017 [32]	Single-center, Double-blind, randomized, placebo-controlled	Mild/moderate proctitis/left colitis/pancolitis	Curcumin (capsule) 450 mg/day + 2.4 g 5-ASA/day (*n* = 29) or placebo + 2.4 g 5-ASA/day (*n* = 33)	UCDAI and endoscopic Baron score evaluation assessed at baseline, and at 4 and 8 weeks	21 participants did not complete the study. No significant difference between the clinical remission and endoscopic remission rates of curcumin and placebo groups (UCDAI).
		*n* = 62 (41♂/21♀)			
India		24–48 years	8 weeks		
Sadeghi et al. 2019 [33]	Double-blind, randomized, placebo-controlled	Mild/moderate proctitis/left colitis/pancolitis)	Curcumin (capsule-turmeric extract) 1.500 mg/day + routine drugs (*n* = 35) or placebo + routine drugs (*n* = 35)	SCCAI, IBDQ-9, ESR, hs-CRP, anthropometric indices and dietary intakes were assessed at baseline and after 8 weeks	4 patients curcumin group and 3 from placebo group withdrew from the study. Clinical remission 83.9% curcumin vs. 43.8% placebo group. Significant reduction hs-CRP concentrations in Curcumin compared to placebo group. Significant decrease of ESR in curcumin group. Significant increase of mean of IBDQ-9 score in curcumin group compared to placebo group.
		*n* = 70 (21♂/49♀)			
Iran		27–53 years	8 weeks		

LEGEND: 5-ASA—mesalamine; UC—ulcerative colitis; SCCAI—simple clinical colitis activity index; UCDAI—ulcerative colitis disease activity index, IBDQ-9—Inflammatory Bowel Disease Questionnaire; ESR—erythrocyte sedimentation rate; hs-CRP—high-sensitivity C-reactive protein; SAE—serious adverse effects.
